# Supplementary material for: Clinical Presentation Is Dependent on Age and Calendar Year of Diagnosis in Celiac Disease: A Hungarian Cross-Sectional Study
Source: J Pers Med. 2023 Mar 8;13(3):487. doi: 10.3390/jpm13030487 (PMC10054661; doi:10.3390/jpm13030487)
Supplement: Supplementary file 1 [file jpm-13-00487-s001.zip › jpm-2258172-supplementary.pdf]

Supplementary Table S1. Data quality of the variables analyzed

Variable	Data quality (available% of total)
Age at diagnosis	738/738 (100)
Calendar year of diagnosis	738/738 (100)
Gender	738/738 (100)
Clinical presentation at diagnosis	738/738 (100)
Serology at diagnosis - tTG IgA	605/738 (82)
Serology at diagnosis - tTG IgG	583/738 (79)
Serology at diagnosis - EMA IgA	587/738 (80)
Serology at diagnosis - EMA IgG	412/738 (56)
Histology at diagnosis	543/738 (73)

tTG: anti-tissue transglutaminase antibody, EMA: anti-endomysial antibody.
